# Detection of CTLA-4 level and humeral immune response after the second dose of COVID-19 vaccine in certain Iraqi provinces participants

**DOI:** 10.1371/journal.pone.0296521

**Published:** 2024-01-05

**Authors:** Laith A. I. K. Al-Kaif, Hussain Al-Ameri, Wael Rasheed Obaead Alfatlawi, Ammar Eesa Mahdi, Younis A. K. Al-Khafaji, Mohammad Abd-Kadhum Al-Saadi, Alaa H. Al-Charrakh, Raheem T. Al-Mammori, Mohammed Ahmed Al-Kaif

**Affiliations:** 1 Department of Medical Microbiology, Hammurabi College of Medicine, University of Babylon, Hillah, Babylon, Iraq; 2 Department of Medical Laboratory Techniques, Al-Mustaqbal University, Hillah, Babylon, Iraq; 3 Department of Microbiology, College of Medicine, Babylon University, Hillah, Babylon, Iraq; 4 Basic Science Department, College of Dentistry, University of Babylon, Hillah, Babylon, Iraq; 5 Department of Anesthesia Techniques, Al-Mustaqbal University, Hillah, Babylon, Iraq; 6 Babylon GIT Diseases Center, Merjan Medical City, Babylon Province, Hilla, Iraq; 7 Department of Cardiology, Qingpu Branch of Zhongshan Hospital Affiliated to Fudan University, Shanghai, China; Pasteur Institute of Iran, ISLAMIC REPUBLIC OF IRAN

## Abstract

**Background:**

Evaluating immune responses following COVID-19 vaccination is paramount to understanding vaccine effectiveness and optimizing public health interventions. This study seeks to elucidate individuals’ immune status after administering a second dose of diverse COVID-19 vaccines. By analyzing immune responses through serological markers, we aim to contribute valuable insights into the uniformity of vaccine performance.

**Methods:**

A total of 80 participants were enrolled in this study, with demographic and COVID-19 infection-related data collected for categorization. Serum samples were acquired within a specified timeframe, and SARS-CoV-2 IgM/IgG rapid tests were conducted. Moreover, CTLA-4 levels were measured through ELISA assays, allowing us to assess the immune responses comprehensively. The participants were divided into eight groups based on various factors, facilitating a multifaceted analysis.

**Results:**

The outcomes of our investigation demonstrated consistent immune responses across the diverse types of COVID-19 vaccines administered in Iraq. Statistical analysis revealed no significant distinctions among the vaccine categories. In contrast, significant differences were observed in CTLA-4 among the control group (non-infected/non-vaccinated, infected/non-vaccinated) and infected/Pfizer, non-infected/Pfizer, and infected/Sinopharm, non-infected/sinopharm (P = 0.001, < 0.001, 0.023, respectively). This suggests that these vaccines exhibit comparable effectiveness in eliciting an immune response among the study participants.

**Conclusions:**

In conclusion, our study’s results underscore the lack of discriminatory variations between different COVID-19 vaccine types utilized in Iraq. The uniform immune responses observed signify the equitable efficacy and performance of these vaccines. Despite minor quantitative discrepancies, these variations do not hold statistical significance, reaffirming the notion that the various vaccines serve a similar purpose in conferring protection against COVID-19.

## Introduction

The global spread of the COVID-19 pandemic, caused by the coronavirus disease 2019 (COVID-19), began after the first case of this infection was recorded in the Chinese city of Wuhan in December 2019. It began to spread rapidly throughout the world, including Iraq, resulting in a substantial loss of life across the globe. The COVID-19 pandemic has become a major global health problem [1, 2]. Patients with SARS-CoV-2 pneumonia have been associated with acute coronary syndromes. Furthermore, all subjects received dual antiplatelet therapy [3–5]. Akşit E. suggested using ticagrelor in a patient suffering from myocardial infarction during the epidemic for three reasons: (1) Due to its pleiotropic effects, there is less risk due to lower levels of pro-inflammatory markers and suppressed suppression. Activation of platelets via adenosine A2A and A2B receptors, which reduces the chance of intravascular coagulation; (2) ticagrelor has shown the potential to reduce thromboinflammatory biomarkers; and (3) recent research shows that it has antibiotic potential against Gram-positive bacteria, which may increase the chances of survival in patients with coexisting diseases [6–8].

Consequently, the scientific community faced an urgent imperative to develop vaccinations globally. The World Health Organization (WHO) outlined the distribution plan for COVID-19 vaccinations, setting the timeline for September 2020 [9]. The COVID-19 vaccines were developed using a variety of methods, such as mRNA (Modera and Pfizer), adenoviral vector (Johnson & Johnson and AstraZeneca), inactivated whole-virus vaccines (Sinopharm), and subunit vaccinations (Novavax (USA)). However, only three vaccines—made by Pfizer, AstraZeneca, and Sinopharm—have been applied in Iraq [10]. Emergency utilization authorization was granted for the mRNA vaccine "Pfizer BioNTech" on December 31, 2020, and subsequently for the adenoviral vector vaccines ChAdOx1 nCoV-19 (AstraZeneca-Oxford) on February 15, 2021. The respective efficacy rates for these vaccines were reported at 95% and 70%. Concurrently, the inactivated SARS-CoV-2 vaccine "BBIBP-CorV" by Sinopharm (Beijing, China) exhibited a seroconversion rate of 92% to 100% with no associated risks. Notably, Sinopharm’s Vaccine emerged as the initial and well-tolerated option for vaccination among the Iraqi population [11–14]. According to Teijaro and Farber [10], each requires the SARS-CoV-2 native viral spike protein (S) to elicit neutralizing solid antibodies. After vaccination, memory T and B cells specific for the S protein develop and circulate, working together to stop additional SARS-CoV-2 infection [15]. The Ministry of Health (MOH) in Iraq documented a cumulative count of 2,325,522 confirmed COVID-19 cases, resulting in 25,213 deaths attributed to SARS-CoV-2 infection. These cases were recorded from the initial report of the first case on February 24, 2020, through May 7, 2022. Conversely, 10,538,065 individuals were vaccinated using three distinct COVID-19 vaccines as part of the vaccination program until May 7, 2022. The vaccination rate averaged between 1,000 and 15,000 individuals per day, with only 25% of the population having received vaccination by then [16].

Immunoassay techniques are employed to identify and quantify antigen-antibody interactions [17]. These methods offer valuable insights into the dynamics of virus infections and prior exposures [18, 19]. Unlike viral RNA, antibodies demonstrate higher resilience to degradation and are less influenced by transportation, storage, and selection [20]. In the context of microbial infections, the production of IgM serves as the initial defense, followed by the development of IgG for long-term immunity and immunological memory [17]. IgM and IgG antibodies have been detected in patient blood samples within three to six days and eight days following SARS infection [21–23]. Therefore, the presence of these antibodies aids in estimating the infection date. There are SARS-CoV2-specific IgG and IgM antibodies, and they can be detected within 3–4 days post-symptom onset [18]. Presently, serodiagnosis of SARS-CoV-2 infection in clinical microbiology laboratories primarily employs antibody detection through indirect immunofluorescence assays and enzyme-linked immunosorbent assays (ELISA) using cell culture extract [24, 25]. While IgM antibodies can emerge as swiftly as viral genetic material in the respiratory tract, their development timing (four days to 10–14 days after symptom onset) restricts their utility in acute-phase diagnosis [26, 27]. Therefore, serological tests that detect specific SARS-CoV-2 antibodies in patient blood samples serve multiple purposes, including patient follow-up, serological surveillance, and identification of previously exposed individuals [28]. Additionally, these serological assessments prove effective in evaluating vaccine efficacy [29].

There are two categories of immunological responses: innate and adaptive immune responses [30]. The stimulation of TLRs by interactions with ligands triggers an intracellular downstream signaling cascade that serves as the innate immunological, activating the host defense system [31]. Following viral replication, TLR3 detects the viral dsRNA, which causes TRIF-mediated inflammatory signaling to be triggered. TLR7/8 recognizes SARS-ssRNA CoV-2. Target genes, such as types I and III IFNs and other essential pro-inflammatory cytokines, are expressed more favorably [31].

Around a week after the onset of symptoms, adaptive immune responses (both T and B cells) against SARS-CoV-2 begin to show. T cells provide two primary purposes: While CD4+ T cells excite both B cells and CD8+ T cells as well as create cytokines that help in the recruitment of immune cells, CD8+ T cells actively target and kill virus-infected cells [32]. T helper cells help B cells transform into plasma cells, which then produce antibodies (Abs) termed neutralizing antibodies that are directed against a viral antigen (Ag). SARS-CoV’s antibody profile produces IgM and IgG, and seroconversion—which is mediated by helper T cells—has been discovered later on. The helper T cell also plays a role in isotype switching [32].

Because so many CD8+ infiltrating cells (80%) were drawn to the infection site, it was found that cellular immunity played a significant part in protecting against SARS-CoV-2 [33]. According to recent research, certain IgG-neutralizing antibodies that target the receptor-binding domain (RBD) of the spike protein can successfully disrupt the fusion of the virus with ACE2 receptors, preventing viral entrance into lung cells and continued transmission [34]. They looked at the characteristics of CD4+ and CD8+ T-cell immune responses in a study that included verified COVID-19 cases. They noticed that the spike protein activated 100% of CD4+ T cells and that the anti-SARS-CoV-2 IgG and IgA titers were related to the strength of the spike protein response [35].

CTLA-4, a surface-receptive immunoglobulin cell, is a T-cell activation inhibitor [36]. It is expressed predominantly on naive T cells after activation [37]. CTLA-4 is a high-affinity CD28 homolog for B7- 1/2. Although the interaction of the CD 28:B7- 1/2 serves as a co-simulator for T-cell propagation and activation, CTLA-4:B7- 1/2 binding, it is also a coinhibitory signal to stop early T-cell activation, leading to inhibition of Tcell costimulation [38, 39].

The interaction between a T cell and an antigen-presenting cell (APC) involves a delicate balance between signal strength, quality, and duration, and CTLA-4 largely governs this balance [40]. Studies have demonstrated that blocking CTLA-4 with specific antibodies enhances T-cell immune responses, improving disease progression, pathogen elimination, and heightened survival rates in septic and immunodeficient patients [41, 42]. The collaborative action of CTLA-4 and B7 has been identified as the reason behind the inhibition of T lymphocyte function. Consequently, targeting the CTLA-4/B7 pathways amplifies T-cell activity, aiding in the immune-based detection of tumors. In summary, the CTLA-4/B7 pathways play a pivotal role in COVID-19 infection, underscoring the need for strategies to combat this viral infection more effectively [43]. This research evaluates the immune responses following COVID-19 vaccination, which is paramount to understanding vaccine effectiveness and optimizing public health interventions. This study seeks to elucidate individuals’ immune status after administering a second dose of diverse COVID-19 vaccines. By analyzing immune responses through serological markers, we aim to contribute valuable insights into the uniformity of vaccine performance.

## Materials and methods

### Study design and participants

This case-control study was conducted within the Department of Medical Laboratory Techniques at Al-Mustaqbal University College from November 1, 2021, to March 23, 2022. A uniform questionnaire was administered to all Iraqi participants before collecting blood samples. Eighty blood specimens were procured and categorized into eight distinct groups, each comprising 10 participants (Non-infected / Non-vaccinated, Infected / Non-vaccinated, Pfizer / Non-infected, Pfizer / Infected, AstraZeneca / Non-infected, AstraZeneca / Infected, Sinopharm/Non-infected, and Sinopharm / Infected). Individuals with cancer, immunosuppressive or chemotherapeutic patients, any patients experiencing acute or chronic inflammation or infection, and pregnant women were not allowed to participate in the study. The questionnaire encompassed essential demographic information such as gender, age, place of residence, history of COVID-19 infection, severity of infection, and the specific type of vaccination received.

## Serological assay

### SARS-CoV-2 IgG/IgM rapid antibody test

Participants’ SARS-CoV-2 status, whether positive or negative, was ascertained through a rapid antibody test kit (ACON Biotech, Hangzhou, China, Address: No. 210 Zhenzhong Road, West Lake District). The test parameters were evaluated in strict adherence to the manufacturer’s instructions, or in cases where participants had been previously infected and confirmed using PCR, CT, CRP/IL-6/D-Dimer/Ferritin, certain results were considered as per their responses.

### Human CTLA-4 ELISA kit

A sandwich ELISA Kit detected the Human Cytotoxic T lymphocyte-associated antigen 4 (CTLA-4) (Bioassay Technology Laboratory, Jiaxing, Zhejiang, China). Parameters are measured according to the instructions of the manufacturing company.

### Statistical analysis

The SPSS software, version 22, was used for the statistical analysis. The Mann-Whitney U and Kruskal-Wallis H tests were used to compare the means of the different groups and to show the data as number (n), percentage, mean, and standard deviation. A statistically significant difference was one with a P value of 0.05.

## Results

### Database characteristics

Table 1 provides an overview of the study’s demographic characteristics. Eighty volunteers in all were enrolled, and they were split into two groups (with and without the SARS-CoV-2 infection). They all had a normal BMI and were non-smokers.

**Table 1:** Study of the population characteristics from the database.

Variables	Number (%)
**Sample Size**	80 (100.0)
**Gender**	
Male	59 (73.8)
Female	21 (26.2)
**Age groups**	
≤ 30	52 (65.0)
31–60	25 (31.2)
≥ 60	3 (3.8)
**Residence**	
Babylon	22 (27.5)
Al-Muthanna	29 (36.2)
Dhi Qar	2 (2.5)
Al-Najaf	12 (15.0)
Karbala	11 (13.8)
Al-Qadisiyah	4 (5.0)
**COVID-19 infection history**	
Yes	40 (50.0)
No	40 (50.0)
**Vaccine type**	
Pfizer	20 (25.0)
AstraZeneca	20 (25.0)
Sinopharm	20 (25.0)
Not-vaccinated	20 (25.0)
**Post-infection duration (Months)**	
5–10	5 (6.2)
11–15	20 (25.0)
16–20	8 (10.0)
21–25	7 (8.8)
**Duration period after 2**^**nd**^ **dose of vaccination (Months)**	
0.5–3	33 (41.2)
4–6	22 (27.5)
7–9	2 (2.5)
10–12	3 (3.8)
**Severity of infection**	
Severe	15 (18.8)
Non-severe	25 (31.2)
**Diabetic**	
Yes	3 (3.8)
No	77 (96.2)

### Viral serodiagnosis

The results of the COVID-19 test used to diagnose SARS-CoV-2 antibodies in all volunteers. None volunteers (0%) had positive COVID-19-IgM results, 40 out of 80 volunteers (50%) had positive COVID-19-IgG results, and zero volunteers (0%) had positive COVID-19-IgM and IgG results. Regarding the other volunteers, the results revealed that 50% of them (or 40 out of 80) tested negative for COVID-19-IgM and IgG antibodies.

## Immune markers of cellular response

### Cytotoxic T- lymphocyte- associated antigen- 4 for SARS-CoV-2

The results in Table 2 showed that there was no significant difference among the individuals with or without SARS-CoV-2 infection. Whereas a very significant difference was observed between SARS-CoV-2 vaccinated and not-vaccinated individuals. There were (59) 73.8% males and (21) 26.2% females among the total (80) volunteers who participated in the study, with no statistically significant differences between males and females. The volunteers observed in this study represented 6 Iraqi governorates as follows: Babylon 22 (27.5%), Al-Muthanna 29 (36.2%), Dhi Qar 2 (2.5%), Al-Najaf 12 (15.0%), Karbala 11 (13.8%), and Al-Qadisiyah 4 (5.0%). As a result, no statistically significant differences among different regions. Also, the results recorded very high significant differences between the individuals vaccinated with the second dose of the different COVID-19 vaccines.

**Table 2:** Summary statistics of CTLA-4 level responses among sera of participants groups.

Variable	Mean ± SD	P value
**SARS-CoV-2 Infection status**		
Positive (n: 40)	20.6 ± 5.1	0.392 *
Negative (n: 40)	23.2 ± 9.7	
**Vaccination Status**		
Vaccinated (n: 60)	23.4 ± 8.4	< 0.001*
Not-vaccinated (n: 20)	17.2 ± 2.3	
**Gender**		
Male (n: 59)	22.2 ± 8.4	0.603 *
Female (n: 21)	20.8 ± 5.9	
**Residence**		
Babylon (n: 22)	20.8 ± 5.2	0.199 **
Al-Muthanna (n: 29)	20.5 ± 6.1	
Dhi Qar (n: 2)	20.5 ± 0.1	
Al-Najaf (n: 12)	22.1 ± 11.7	
Karbala (n: 11)	27.8 ± 11.1	
Al-Qadisiyah (n: 4)	20.8 ± 2.01	
**Types of Vaccine**		
Pfizer (n: 20)	22.7 ± 5.2	< 0.001**
Astrazeneca (n: 20)	22.2 ± 9.5	
Sinopharm (n: 20)	25.4 ± 9.7	
Not vaccinated (n: 20)	17.2 ± 2.3	

*Mann-Whitney test,

** Kruskal-Wallis H test, Level of significance is P < 0.05.

Regarding age groups, the comparison between the two groups of individuals in this study showed no significant differences between non-infected individuals and the infected SARS-CoV-2. (P < 0.05) in the mean serum level of CTLA-4 (Table 3).

**Table 3:** Level of CTLA-4 among sera of participants groups with or without COVID-19 according to age groups.

Age groups (years)	SARS-CoV-2 Infection history	
	Positive (n: 40)	Negative (n: 40)
	Mean ± SD	Mean ± SD
**≤ 30**	23.4 ± 9.0	20.1 ± 3.4
**31–60**	23.7 ± 12.9	21.4 ± 7.3
**≥ 60**	20.0 ± 1.2	-
**P value**	0.812	0.865

*Mann-Whitney test, Level of significance is P < 0.05.

Table 4 shows the variables of vaccine types, where no significant differences appeared when comparing three vaccines used in Iraq.

**Table 4:** Comparisons of the vaccine types after the COVID-19 vaccine among sera of participants groups according to CTLA-4 level.

Parameter	Types of Vaccine		Mean ± SD
**CTLA-4**	Pfizer	N: 20	22.7 ± 5.2
	Astrazeneca	N: 20	22.2 ± 9.5
	Sinopharm	N: 20	25.4 ± 9.7
	Total	N: 60	23.4 ± 8.4

In this study, 80 sera samples were screened by ELISA for CTLA-4 for individuals SARS-CoV-2 infected/non-infected, vaccinated/non-vaccinated, and both it. The results in Table 5 showed a significant difference among the control group (Non-infected/Non-vaccinated, Infected/Non-vaccinated) and Infected/Pfizer, Non-infected/Pfizer, and infected /Sinopharm, Non-infected /Sinopharm (P = 0.001, < 0.001, 0.023, respectively) in comparison with group Non-infected/ AstraZeneca, Infected/AstraZeneca (P = 0.14). Thus, the results showed that there were very high significant differences between the vaccinated and non-infected individuals (P < 0.001) compared to the infected and non-vaccinated individuals (P = 0.318).

**Table 5:** Level of CTLA-4 among sera of participants groups with or without COVID-19 according to type of vaccine.

Age groups (years)	SARS-CoV-2 Infection history		P value
	Positive (n:10)	Negative (n:10)	
	Mean ± SD	Mean ± SD	
**Pfizer**	19.1 ± 2.0	26.2 ± 5.1	< 0.001
**AstraZeneca**	22.4 ± 5.4	21.9 ± 12.8	0.14
**Sinopharm**	21.7 ± 8.0	29.1 ± 10.3	0.023
**Not vaccinated**	19.0 ± 1.9	15.5 ± 1.2	0.001

* Mann-Whitney test, Level of significance is P < 0.05.

## Discussion

Since COVID-19 vaccines were made to control the pandemic, which may be devoid of side effects of manufactured vaccines, and in light of our study to evaluate the work of the three vaccines currently available in Iraq in the infected and not infected with SARS-CoV-2, the current study showed that there were no significant differences between the different types of vaccines used in Iraq. These findings support manufacturers’ use of these vaccines.

According to a study conducted by Menni et al. [44], it is clear that this may be associated with an increase in immunogenicity as a result of vaccination, which in turn increases the immune status of these individuals.

In addition, the study conducted by Larijani et al. [45], explained that a person who is immunized against the Coronavirus (Covid-19) has many more advantages than disadvantages, and late adverse events (AEs) appear to be rare. According to another study conducted by Ramezani et al. [46] about testing the durability of antibodies, as the study showed that antibodies produced by all three groups were viable until day 180. However, in contrast to the BBIP-CorV group, a greater rate of antibody titer was observed in the heterologous regimen. Moreover, no significant adverse event was noted. This is consistent with our current study, which supports the work of the three vaccine companies used in Iraq.

This study can be enhanced by increasing the sample size to ascertain the extent of the correlation. CTLA-4, a surface receptor of immunoglobulins [47], T-cell activation inhibitor Co-receptor cytotoxic T-cell lymphocyte antigen-4 (CTLA-4; CD 152) is a key T-cell proliferation and expansion inhibitor [48], it has damping effect on the activation mechanism and terminates T-cell responses. T-cell tolerance and autoimmunity are necessary to regulate [49]. CTLA-4, the first scientifically targeted immune control point receptor, is found exclusively on T cells where the early stage of T cell activation amplitude is primarily controlled [50].

This study showed no significant differences between individuals with or without the SARS-CoV-2 infection. This result agrees with a study conducted in Iraq by Talib *et al*. [51], where it was stated that there are very significant differences between individuals with mild, moderate, and severe infections compared to those who are not infected.

In the current study on demographic characteristics in terms of age group, gender, and residence, there are no significant differences, and this agrees with both Alameri and Kadhim [52], who explained that the above characteristics were not significantly associated with the immune response.

T-cell activation relies not only on the T-cell receptor (TCR) binding to the antigen provided by the antigen-presenting cell (APC) but also on the existence of the costimulatory second signal, usually by binding the CD28 displayed on the T-cell to the CD80/86 located on the APC, the loss of this secondary signal will lead to a T-cell being identified the presented peptide as a "self-antigen" or developing antigen-tolerance [53].

TCR signaling immediately up-regulates CTLA-4 expression on the cell surface, reaching peak expression 2 to 3 days post-stimulation [54], providing a negative feedback loop upon activation of T-cells. CTLA-4 inside the intracellular vesicles is rapidly transferred to the immunological synapse after T-cell activation [55, 56].

CTLA-4 is stabilized with the CD80/CD86 binding in the immune synapse, allowing the CD28 binding to be collected and inhibited. CTLA-4 restricts CD28 signalization downstream and inhibits the pathway PI3K and AKT [57] (de Araújo et al., 2021).

CTLA-4 also removes CD28 ligands CD80/86 from adjacent cells by trans endocytosis in vivo, like APCs, besides inhibiting T-cell activation [58]. Physiologically, CTLA-4 is known mainly to play a modulatory role in T-cell priming in local secondary lymphoid organs by suppressing T-cell activation and preventing T-cell progression [59].

CTLA-4 was among the first and most thoroughly studied immune system co-inhibitor receptors [60]. Given the significance of CTLA-4 for autoimmunity and anti-tumor immunotherapy, the precise pathways responsible for its function still need to be determined. Much controversy has centered on whether CTLA-4 inhibits T-cell response by extrinsic or intrinsic cell mechanisms [61].

Cell-intrinsic mechanisms will represent the direct effects of the co-receptor on the expression cell. In contrast, cell-extrinsic effects are linked to the modulation of activity by the distal cell or cytokine. Both pathways have been involved in the in vivo activity of CTLA-4 [62]. Cell-intrinsic and non-cell autonomous activities of CTLA-4 have also been reported to preserve T-cell tolerance to self-antigenic activity [63].

Therefore, the outcomes of the viral infection vary according to the vigor of the immune response, a process that is regulated by several molecules, including the cell surface receptor CTLA-4 [64], which is consistent with its emerging role in the T regulatory cells in the pathogenesis of the disease.

The immune response via cellular immunity creates superior resistance to viral mutations by cytotoxic T-cells that provide long-term cellular immune protection, which is currently the greatest threat to the global vaccination campaign [65].

Adeno-based COVID-19 vaccines that encode the Spike protein were co-delivered with Ad-9D9 in a recent study by Morena et al. [66], and it was found that this combination produced stronger cellular and humoral immune responses. Comparatively, when the vaccination was combined with the same anti-CTLA-4 in its proteinaceous form, a meager adjuvant effect was obtained. Importantly, the immunostimulatory effect is eliminated when the adjuvant vector is administered at several vaccination vector sites. As Ad-CTLA-4 enhanced the immune response and effectiveness of an Adenovirus-based poly epitope vaccination encoding tumor neoantigens, we demonstrated that Ad-CTLA-4’s adjuvant activity is independent of the Vaccine antigen. As a result, adding an Adenovirus Encoded Adjuvant (AdEnA) to an Adeno-encoded antigen vaccination improves immune responses to viral and tumor antigens. This is a powerful strategy for creating genetic vaccinations that work better.

The research done by Aiello et al. [67], revealed for the first time that the majority of RA patients who underwent a strategy of temporary suspension of immunosuppressive treatment during vaccine administration had antibody-specific and whole-blood spike-specific T-cell responses induced by the COVID-19 mRNA-vaccine. However, the immunosuppressive medication used impacted how strong the individual responses were. The BNT162b2 vaccination proved safe, and RA patients’ disease activity remained steady.

According to studies, the inhibition of CTLA-4 during viral infection or model antigen vaccination enhanced the growth of germinal center B-cells. This was observed during HIV VLP immunization and led to an increase in CD4+ T-cell activation, promoted the growth of Tfh cells specific for the HIV envelope (Env), and markedly increased HIV Gag- and Env-specific IgG with higher avidity and antibody-dependent cellular cytotoxicity (ADCC) abilities. Enhanced levels of class-switched Env- and Gag-specific IgG imply enhanced polyclonal B-cell activation, exhibiting the ability to induce and accelerate ADCC, even if no discernible rise in neutralizing antibodies was seen. Thus, the results of Lewis et al. [68], demonstrated that CTLA-4 inhibition, in combination with a clinically relevant HIV VLP-based vaccination, can boost HIV antigen-specific B-cell and antigen-specific Tfh cell activity levels and influence humoral immune responses.

### Limitation of this study

Due to a number of limitations in our study, we urge caution against interpreting the results too quickly. There was a tiny sample size. Following immunization, each participant’s IgG concentration must be monitored with the first, second, and booster doses. Additionally, only three COVID-19 vaccination types were included in this investigation.

## Conclusions

The accumulated outcome data indicated non-discrimination and the legalization of the use of some of these vaccines because they all perform the same role and have the same effectiveness, with simple arithmetic differences that are not significant.

## Supporting information

S1 Table Characteristics enrolled for eighty volunteers in this study. (DOC)

S1 File (XLS)
