# Supplementary material for: Detection of CTLA-4 level and humeral immune response after the second dose of COVID-19 vaccine in certain Iraqi provinces participants
Source: PLoS One. 2024 Jan 5;19(1):e0296521. doi: 10.1371/journal.pone.0296521 (PMC10769031; doi:10.1371/journal.pone.0296521)
Supplement: S1 Table — (DOC) [file pone.0296521.s001.doc]

S1 Table: Characteristics enrolled for eighty volunteers in this study.

| **No. of samples** | **Sex** | **Age range** | **Residant** | **SARS-COV-2 Infected or not** | **Infected peroid** | **Severe infected** | **Vaccinated or not** | **Vaccinated peroid** | **Type of vaccination** | **Diseases** | **CTLA-4** |
| --- | --- | --- | --- | --- | --- | --- | --- | --- | --- | --- | --- |
| 1 | F | 20 | Al-Muthanna | NOT |  |  | NOT |  |  |  | 14.892782 |
| 2 | F | 20 | Al-Muthanna | NOT |  |  | NOT |  |  |  | 15.948176 |
| 3 | F | 21 | Al-Muthanna | NOT |  |  | NOT |  |  |  | 16.534506 |
| 4 | F | 25 | Al-Muthanna | NOT |  |  | NOT |  |  |  | 15.127314 |
| 5 | M | 26 | Al-Muthanna | NOT |  |  | NOT |  |  |  | 14.518311 |
| 6 | M | 47 | Al-Muthanna | NOT |  |  | NOT |  |  |  | 18.352129 |
| 7 | M | 30 | Al-Muthanna | NOT |  |  | NOT |  |  |  | 15.127314 |
| 8 | F | 20 | Al-Muthanna | NOT |  |  | NOT |  |  |  | 14.775516 |
| 9 | F | 20 | Al-Muthanna | NOT |  |  | NOT |  |  |  | 14.130553 |
| 10 | F | 24 | Al-Muthanna | NOT |  |  | NOT |  |  |  | 15.889543 |
| 11 | M | 18 | Dhi Qar | YES | 21 M | NOT | NOT |  |  |  | 20.52155 |
| 12 | M | 20 | Dhi Qar | YES | 11 M | YES | NOT |  |  |  | 20.404284 |
| 13 | M | 22 | Babylon | YES | 9 M | NOT | NOT |  |  |  | 17.355368 |
| 14 | M | 23 | Babylon | YES | 12 M | NOT | NOT |  |  |  | 18.469395 |
| 15 | M | 22 | Babylon | YES | 6 M | NOT | NOT |  |  |  | 16.241341 |
| 16 | M | 34 | Babylon | YES | 16 M | NOT | NOT |  |  |  | 17.648533 |
| 17 | M | 30 | Al-Najaf | YES | 11 M | NOT | NOT |  |  |  | 19.055725 |
| 18 | M | 25 | Karbala | YES | 12 M | NOT | NOT |  |  |  | 17.00357 |
| 19 | M | 27 | Babylon | YES | 13 M | NOT | NOT |  |  |  | 21.69421 |
| 20 | M | 39 | Al-Muthanna | YES | 11 M | NOT | NOT |  |  |  | 21.10788 |
| 21 | F | 21 | Al-Muthanna | NOT |  |  | YES | 14 D | pfizer |  | 24.156796 |
| 22 | M | 23 | Al-Muthanna | NOT |  |  | YES | 4 D | pfizer |  | 21.870109 |
| 23 | M | 24 | Al-Muthanna | NOT |  |  | YES | 3 M | pfizer |  | 33.596709 |
| 24 | M | 56 | Al-Muthanna | NOT |  |  | YES | 6 M | pfizer |  | 21.811476 |
| 25 | F | 19 | Al-Muthanna | NOT |  |  | YES | 3 M | pfizer |  | 33.244911 |
| 26 | F | 25 | Al-Muthanna | NOT |  |  | YES | 1 M | pfizer |  | 33.127645 |
| 27 | F | 25 | Al-Muthanna | NOT |  |  | YES | 29 D | pfizer |  | 25.563988 |
| 28 | M | 30 | Al-Muthanna | NOT |  |  | YES | 4 M | pfizer |  | 21.576944 |
| 29 | M | 28 | Al-Muthanna | NOT |  |  | YES | 7 M | pfizer |  | 25.153557 |
| 30 | F | 25 | Al-Muthanna | NOT |  |  | YES | 5 M | pfizer | Diabetic | 22.28054 |
| 31 | M | 39 | Al-Najaf | NOT |  |  | YES | 6 M | astrazeneca |  | 14.130553 |
| 32 | M | 41 | Al-Najaf | NOT |  |  | YES | 3 M | astrazeneca |  | 57.636239 |
| 33 | M | 41 | Al-Najaf | NOT |  |  | YES | 4 M | astrazeneca |  | 19.055725 |
| 34 | M | 67 | Al-Najaf | NOT |  |  | YES | 2 M | astrazeneca |  | 18.879826 |
| 35 | M | 61 | Al-Najaf | NOT |  |  | YES | 2 M | astrazeneca |  | 21.166513 |
| 36 | M | 54 | Al-Najaf | NOT |  |  | YES | 6 M | astrazeneca |  | 18.586661 |
| 37 | M | 37 | Al-Najaf | NOT |  |  | YES | 6 M | astrazeneca |  | 15.361846 |
| 38 | M | 61 | Al-Najaf | NOT |  |  | YES | 6 M | astrazeneca |  | 19.876587 |
| 39 | M | 47 | Al-Najaf | NOT |  |  | YES | 6 M | astrazeneca |  | 18.645294 |
| 40 | M | 45 | Al-Najaf | NOT |  |  | YES | 6 M | astrazeneca |  | 15.655011 |
| 41 | M | 24 | Karbala | NOT |  |  | YES | 4 M | sinopharm |  | 21.69421 |
| 42 | M | 23 | Babylon | NOT |  |  | YES | 4 M | sinopharm |  | 30.665059 |
| 43 | M | 23 | Babylon | NOT |  |  | YES | 2 M | sinopharm |  | 21.10788 |
| 44 | M | 26 | Karbala | NOT |  |  | YES | 2 M | sinopharm |  | 21.459678 |
| 45 | F | 22 | Babylon | NOT |  |  | YES | 3 M | sinopharm |  | 20.697449 |
| 46 | F | 24 | Babylon | NOT |  |  | YES | 3 M | sinopharm |  | 22.163274 |
| 47 | M | 28 | Karbala | NOT |  |  | YES | 2 M | sinopharm |  | 46.671868 |
| 48 | M | 29 | Karbala | NOT |  |  | YES | 1 M | sinopharm |  | 45.264676 |
| 49 | M | 33 | Karbala | NOT |  |  | YES | 3 M | sinopharm |  | 36.710405 |
| 50 | M | 35 | Karbala | NOT |  |  | YES | 3 M | sinopharm | Diabetic | 24.567227 |
| 51 | M | 45 | Babylon | YES | 15 M | YES | YES | 5 M | pfizer |  | 20.169752 |
| 52 | M | 24 | Babylon | YES | 16 M | YES | YES | 2 M | pfizer |  | 16.944937 |
| 53 | M | 23 | Babylon | YES | 24 M | YES | YES | 2 M | pfizer |  | 16.241341 |
| 54 | F | 22 | Babylon | YES | 12 M | YES | YES | 1 M | pfizer |  | 17.648533 |
| 55 | F | 23 | Babylon | YES | 24 M | YES | YES | 12 M | pfizer |  | 19.055725 |
| 56 | M | 21 | Babylon | YES | 15 M | YES | YES | 2 M | pfizer |  | 17.00357 |
| 57 | M | 26 | Babylon | YES | 24 M | YES | YES | 3 M | pfizer |  | 21.69421 |
| 58 | M | 27 | Babylon | YES | 21 M | YES | YES | 11 M | pfizer |  | 21.10788 |
| 59 | M | 25 | Babylon | YES | 21 M | YES | YES | 2 M | pfizer |  | 20.169752 |
| 60 | F | 25 | Babylon | YES | 24 M | YES | YES | 1 M | pfizer |  | 21.342412 |
| 61 | M | 20 | Al-Qadisiyah | YES | 12 M | NOT | YES | 3 M | astrazeneca |  | 20.990614 |
| 62 | F | 23 | Al-Najaf | YES | 14 M | NOT | YES | 5 M | astrazeneca |  | 27.440244 |
| 63 | M | 23 | Karbala | YES | 17 M | NOT | YES | 6 M | astrazeneca |  | 18.586661 |
| 64 | M | 22 | Al-Qadisiyah | YES | 12 M | NOT | YES | 6 M | astrazeneca |  | 20.697449 |
| 65 | M | 25 | Babylon | YES | 9 M | NOT | YES | 5 M | astrazeneca |  | 20.697449 |
| 66 | M | 26 | Al-Qadisiyah | YES | 14 M | NOT | YES | 4 M | astrazeneca |  | 18.352129 |
| 67 | M | 31 | Al-Qadisiyah | YES | 14 M | NOT | YES | 4 M | astrazeneca |  | 23.277301 |
| 68 | M | 33 | Karbala | YES | 17 M | NOT | YES | 11 M | astrazeneca |  | 35.531598 |
| 69 | M | 35 | Karbala | YES | 17 M | NOT | YES | 4 M | astrazeneca |  | 21.342412 |
| 70 | M | 46 | Karbala | YES | 14 M | NOT | YES | 3 M | astrazeneca |  | 17.296735 |
| 71 | M | 32 | Babylon | YES | 17 M | NOT | YES | 9 M | sinopharm |  | 39.811807 |
| 72 | F | 38 | Al-Muthanna | YES | 9 M | YES | YES | 2 M | sinopharm |  | 18.997092 |
| 73 | M | 23 | Al-Muthanna | YES | 5 M | NOT | YES | 3 M | sinopharm |  | 28.026574 |
| 74 | M | 31 | Al-Muthanna | YES | 13 M | NOT | YES | 2 M | sinopharm |  | 14.482351 |
| 75 | M | 52 | Al-Muthanna | YES | 11 M | NOT | YES | 3 M | sinopharm |  | 16.475873 |
| 76 | F | 40 | Al-Muthanna | YES | 11 M | NOT | YES | 2 M | sinopharm |  | 15.83091 |
| 77 | F | 25 | Al-Muthanna | YES | 11 M | NOT | YES | 2 M | sinopharm |  | 28.495638 |
| 78 | M | 30 | Al-Muthanna | YES | 16 M | YES | YES | 4 M | sinopharm | Diabetic | 17.062203 |
| 79 | M | 34 | Babylon | YES | 14 M | YES | YES | 1 M | sinopharm |  | 20.462917 |
| 80 | M | 31 | Al-Muthanna | YES | 18 M | YES | YES | 4 M | sinopharm |  | 17.296735 |
